# Monitoring Individual Sleep and Nocturnal Heart Rate Variability Indices: The Impact of Training and Match Schedule and Load in High-Level Female Soccer Players

**DOI:** 10.3389/fphys.2021.678462

**Published:** 2021-04-26

**Authors:** Júlio A. Costa, Pedro Figueiredo, Fábio Y. Nakamura, António Rebelo, João Brito

**Affiliations:** ^1^Portugal Football School, Portuguese Football Federation (FPF), Oeiras, Portugal; ^2^Research Center in Sports Sciences, Health Sciences and Human Development (CIDESD), University Institute of Maia (ISMAI), Maia, Portugal; ^3^Associate Graduate Program in Physical Education UPE/UFPB, João Pessoa, Brazil; ^4^Centre of Research, Education, Innovation and Intervention in Sport (CIFI^2^D), Faculty of Sport, University of Porto, Porto, Portugal

**Keywords:** overnight measurements, parasympathetic system, sleep accelerometer, recovery, women football

## Abstract

**Purpose:**

To describe individual sleep habits and nocturnal heart rate variability (HRV) responses, and to explore intra-individual associations of workload with sleep and nocturnal HRV indices in high-level female soccer players throughout a 2-week competitive period.

**Materials and methods:**

The study followed a descriptive, observational design. Thirty-four high-level female soccer players (aged 20.6 ± 2.3 years) wore wrist actigraph units and heart rate (HR) monitors during night-sleep to record objective sleep and HRV data throughout 14 days [six evening-time training sessions (ET), six rest-days (RD), and two match-days (MD)]. During each ET and MD, exercise HR (HR_exe_), %HR_peak_, training impulse (TRIMP), session rating of perceived exertion (s-RPE) and perceived ratings of wellbeing were monitored.

**Results:**

After ET, a higher number of players (17–22) slept less than 7 h/night, in contrast to the remaining days (i.e., MD and RD), but only 1–6 players had a sleep efficiency < 75%. The coefficient of variation (CV) for sleep duration and sleep efficiency ranged between 9–22% and 2–11%, respectively. A *small* negative within-subject correlation was found between TRIMP and sleep duration [*r* = −0.25 (−0.36; −0.12); *P* < 0.001] and sleep efficiency [*r* = −0.20 (−0.32; −0.08); *P* = 0.004]. A *moderate* and *small* negative within-subject correlation was found between s-RPE and sleep duration [*r* = −0.43 (−0.53; −0.32); *P* < 0.001] and sleep efficiency [*r* = −0.17 (−0.30; −0.05); *P* = 0.02]. Nocturnal HRV for the time-domain analyses ranged from 4.1 (3.9; 4.3) to 4.4 (4.1; 4.6) ln[ms], and for the frequency-domain analyses ranged from 6.3 (5.9; 6.7) to 7.5 (7.1; 7.9) ln[ms^2^]. CV for time-domain HRV ranged from 3 to 23%, and from 4 to 46% for the frequency-domain. Higher CV fluctuations in time- and frequency-domain HRV were particularly observed in four players.

**Conclusion:**

Overall, this study highlights the individual variability of sleep and nocturnal HRV indices, indicating that sleep duration may be affected by training and match schedules and workloads. Training and matches workload were not associated with nocturnal HRV in high-level female soccer players.

## Introduction

Athletes, coaches, and health and performance supporting staff should adopt an evidence-based approach to design and monitor training programs. Appropriate load monitoring is crucial for determining whether a player is adapting to a training program, and minimizing the risk of developing non-functional overreaching, illness, or injury (Kalkhoven et al., 2021). Consequently, attention has been given to the evaluation of monitoring tools that may indicate general signs of fatigue and/or health status of athletes (Peake et al., 2018). These indicators include heart-rate (HR) derived indices (Buchheit, 2014) and sleep (Walsh et al., 2020) monitoring.

Non-invasive time-efficient devices such as wearable actigraphy, to assess sleep duration and quality, and HR monitors, to record heart rate variability (HRV) indices, can provide detailed information about positive and negative adaptions over short and/or long periods throughout the competitive season in athletes (Sargent et al., 2016; Plews et al., 2017). Recently, the use of HRV indices during sleep has been implemented to evaluate exercise-induced disturbances in allostatic load (Michael et al., 2018), as well as recovery from daily workloads and other sources of stress (Costa et al., 2019a). In fact, it is currently accepted that overnight sleep measurements (free of external disruptive events) over several days are appropriate for tracking recovery of HRV following exercise (Al Haddad et al., 2009; Costa et al., 2018c).

The effects of exercise on sleep and overnight HRV indices have been ascribed to factors such as time of day (i.e., training and/or match schedule) (Sargent et al., 2014; Costa et al., 2018a), chronotype (Bonato et al., 2017; Vitale et al., 2017), training, and match load (Buchheit et al., 2004; Costa et al., 2018b), and more recently after an acute session of high-intensity interval training in non-professional male soccer player’s (Bonato et al., 2020). This fact could be relevant because several athletes train or compete during the late evening. For instance, female soccer players may habitually train late in the evening, close to bedtime sleep, due to their daily life commitments (e.g., job, school) which need to be reconciled with training schedules (Costa et al., 2018a,b). In fact, training and match schedule and workload may cause disruption in sleep and/or HRV indices in high-level female soccer players (Costa et al., 2018a,b).

Previous studies examining sleep and nocturnal HRV indices in high-level female soccer players (Costa et al., 2018a,b,c) have typically averaged data across several nights, providing a mean estimate of sleep habits and HRV responses. While such an approach is useful to provide basic insight into sleep and HRV indices in athletes, it lacks details of how these indicators may vary across the days (Nedelec et al., 2018; Munoz-Lopez and Naranjo-Orellana, 2020). For instance, a very large between-night individual variability in sleeping time (Van Dongen, 2005) might indicate the need for individualized sleep education strategies and interventions to promote appropriate sleep (Nedelec et al., 2018). As a result, an increased interest toward individualized approaches has given rise to a variety of athlete-monitoring strategies, enabling coaches to better manage recovery and fatigue, and prescribe training on an individual basis (Chrzanowski-Smith et al., 2019). Actually, this is feasible in elite sports, and individual analysis methods enable to track individual changes in sleep and HRV indices in elite female soccer players (Costa et al., 2019a).

Periods of intensified training loads may increase the level of disturbance associated with sleep (Hausswirth et al., 2014) and HRV (Sekiguchi et al., 2019) indices. However, it is unfortunate that almost no information exists on intra-individual associations between workload, sleep and nocturnal HRV indices in high-level female soccer players. Thus, it is important to use valid load metrics to properly contextualize the impact of training and/or match workload on sleep habits and nocturnal HRV responses (Costa et al., 2018b), especially in women (Okholm Kryger et al., 2021), who are less investigated than men. Training and/or match workload can be quantified with different tools and metrics, including the session rating of perceived exertion (s-RPE), training impulse (TRIMP), peak HR (% HR_peak_), and the average HR during the exercise session (HR_*ex*_) (Halson, 2014). All these metrics have been recently used as valid measures of internal load in high-level female soccer players, and strong intra-individual associations exist between s-RPE and TRIMP (Costa et al., 2019b).

Therefore, the purpose of the current study was to: (1) describe the individual sleep habits and nocturnal HRV responses, and (2) to explore intra-individual associations of training and match load with sleep and nocturnal HRV indices in high-level female soccer players, throughout a 2-week in-season competitive period. We hypothesized that players would present a negative within-subject associations of sleep and nocturnal HRV indices with training and match workload.

## Materials and Methods

### Subjects

Thirty-four high-level outfield (11 attackers, 10 midfielders, seven fullbacks, and six central defenders) female soccer players (age: 20.6 ± 2.3 years; height: 1.6 ± 0.1 m; body mass: 22.1 ± 2.3 kg; mean ± SD) competing in the first division of the Portuguese soccer league were invited and accepted to voluntarily participate in the study. The following exclusion criteria were defined: age ≤ 18, tobacco use, had at least 5 years of soccer experience, and use of medications and medical conditions contraindicating physical exercise as diagnosed by a sports medicine physician. Players were requested to avoid alcohol and caffeine consumption. Data collection was running during the second month (October) of the in-season competitive period. Female soccer players habitually trained three times per week (∼90 min each training session) with a match played every Sunday. The study design was carefully explained to the participants, and written informed consent was obtained. The study followed the Declaration of Helsinki and was approved by the Ethics Committee of the Faculty of Sports, University of Porto (CEFADE 03.2017).

### Study Design

**FIGURE 1:**
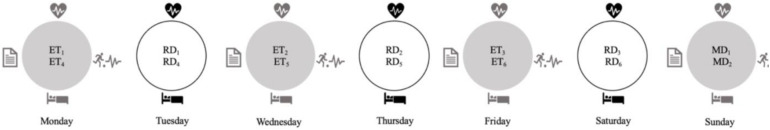
Schematic showing the study design. Heart rate, session-rating of perceived exertion and perceived ratings of wellbeing included evening-time training sessions (ET) and match-days (MD). During night-sleep of the 14 days, including rest-days (RD), sleep and nocturnal heart rate variability indices were assessed using heart rate monitors and accelerometers, respectively.

The study followed a descriptive, observational design, highlighting individual sleep habits and nocturnal HRV responses, as well as intra-individual associations of training and match workload with sleep and nocturnal HRV indices throughout a 2-week in-season competitive period. Data collection was performed throughout 14 days, encompassing six evening-time training sessions (ET); six rest-days [RD, i.e., days without any type of training or exercise) and two match-days (MD)] (Figure 1).

Players sleep and nocturnal HRV indices were monitored every night throughout the observation period. Each player placed HR monitor and wrist-worn accelerometer at home, ∼15 min before going to bed. The players were asked to follow their normal sleep–wake cycle in their own houses to avoid disturbing their regular habits. The study procedures did not constrain the sleeping habits of the players, granting higher ecological validity to the investigation. Each morning, players were instructed to gently remove the electrodes and accelerometers before leaving bed.

Evening-time training session and MD started around 21:00 and 15:00 h, respectively, with approximately 90 min of training and match volume. Training and match workloads were quantified using HR (i.e., HR_exe_ and % HR_peak_), TRIMP and the s-RPE. Players were required to complete a minimum match duration of 60 min in order to have their weekly data included in the analysis (Thorpe et al., 2016). The training sessions proposed by coaches consisted mostly of: soccer practice drills [skill development (such as ball control, dribbling skills, passing accuracy, body control; time spent ∼10 min) and small-sided games (e.g., 4 vs. 4 without goalkeepers; time spent ∼10 min)], resistance training (whole-body workouts involving for example power cleans and squats; time spent ∼10 min) and scrimmages (i.e., 11 vs. 11; time spent ∼60 min).

A psychometric questionnaire (i.e., perceived ratings of wellbeing) was used to assess general indicators of players’ wellness (Hooper et al., 1995). One week before starting the data collection, players completed a Yo-Yo Intermittent Endurance Test—Level 2 (YY-IET2) (Bangsbo, 1994) and HR_peak_ was collected during the test.

During the 5 weeks prior to the study, players were familiarized with the devices used during the investigation to avoid training activities or sleep pattern being disturbed by the equipment. All training sessions and matches were performed on an outdoor artificial turf pitch, which was the usual training venue of the investigated female soccer players.

Ambient temperature ranged from 16 and 18°C during day and 12–14°C during evening. There was no interference by the research team in the athletes’ regular training and match schedule or sleep/wake patterns. Players were requested to avoid alcohol and caffeine consumption during the data collection period.

### Training and Match Load Monitoring

Heart rate data during training sessions and matches were recorded *via* radio telemetry (Firstbeat Sports, Finland) and exported to Firstbeat Sports Server^®^ software (version 4.7.3.1). The software also calculated the HR_*ex*_, expressed in absolute values [beats per min (bpm)], and % HR_peak_.

Training impulse was calculated as proposed by Banister (1991): *TRIMP* = T × [(HR_*ex*_–HR_*rest*_)/(HR_peak_–HR_*rest*_)] × 0.86*e ^1.67^*^[(HRex–HRrest)/(HRpeak–HRrest)]^; where T is the duration of the workout in minutes; HR_*ex*_ the average HR during exercise; HR_*rest*_ the average HR during rest, measured during 5 min of seated rest before starting each training session and match; HR_peak_ was determined as the highest value reached during the YY-IET2 test, and e∼2.718.

Players reported individual RPE using the Borg category ratio scale (CR10) after each training session and match, which was the usual routine of the investigated female soccer players. The CR10 score (perceived intensity) was subsequently multiplied by the individual exposure time (training and match volume), thus providing an overall load quantification of the session or match (i.e., s-RPE) (Foster et al., 2001).

### Perceived Ratings of Wellbeing

Players reported individual wellbeing using the Hooper’s Index scale (Hooper et al., 1995) before each training session and match. The Hooper’s Index scale is a questionnaire involving wellbeing ratings relative to fatigue, stress level, muscle soreness and sleep quality/disorders (scales of 1–7). The Hooper’s Index is the summation of these four ratings.

### Sleep Monitoring

During each night-sleep of the 14 observation days, players worn a 3-axial accelerometer (Actigraph LLC wGT3X-BT, Pensacola, FL, United States) on the non-dominant wrist during each night-sleep. Data were analyzed using proprietary software (ActiLife LLC Pro software v6.13.3, Pensacola, FL, United States). The sampling frequency was 50 Hz and the epoch of activity counts was 60 s (Sadeh et al., 1994). Accelerometer data was extracted using the *Sadeh’s* algorithm, originally validated on a healthy sample of adolescents and young adults (age range 10–25 years) (Sadeh et al., 1994). Sleep indices included sleep duration (amount of sleep in hours) and sleep effciency (percentage of time in bed that was spent asleep) (Sadeh et al., 1994), which were analyzed according to the National Sleep Foundation (Hirshkowitz et al., 2015; Ohayon et al., 2017). A sleep duration <7 h was considered an indicator of inappropriate sleep quantity, and a sleep efficiency ≤ 74% was considered an inappropriate sleep quality (Hirshkowitz et al., 2015; Ohayon et al., 2017).

### Nocturnal HRV Monitoring

Players wore HR monitors (Firstbeat Bodyguard2^®^, Firstbeat Technologies, Finland) to record HRV during night-sleep. Data were analyzed using the slow-wave sleep episode (SWSE) method, which accounts for the deep stage of sleep (Buchheit, 2014). This method records 10 min of normal R-R intervals (Brandenberger et al., 2005). Time domain measure consisted of RMSSD (square root of the mean of the sum of the squares of differences between adjacent NN intervals; vagal modulation index) (Mourot et al., 2004). Fast Fourier Transform [FFT (Welch’s periodogram: 300-s window with 50% overlap)] (TaskForce, 1996) was used to obtain measures of nocturnal HRV in the frequency-domain, considering both low frequency (LF: 0.004–0.15 Hz) and high frequency (HF: 0.15–0.4 Hz) indices (TaskForce, 1996). For frequency analyses, R-R trend components were removed using an advanced smoothness prior approach, with a smoothing parameter of λ = 500, which corresponds to a cut-off frequency of 0.035 Hz (TaskForce, 1996). R-R recordings were exported using the Kubios version 3.2 Heart Rate Variability software (Biosignal Analysis and Medical Imaging Group at the Department of Applied Physics, University of Kuopio, Kuopio, Finland) (Tarvainen et al., 2014).

### Statistical Analysis

Sample distribution was tested using the Shapiro–Wilk test for sleep (i.e., sleep duration and efficiency) and nocturnal HRV indices (lnRMSSD, lnLF, and lnHF), training and match load parameters (HR_*ex*_, HR_peak_, TRIMP, and s-RPE) and perceived rating of wellbeing data, for each day of the observation period. Variables are presented as mean with the 95% confidence interval (CI) unless otherwise stated. The coefficient of variation [CV; CV = (standard deviation/mean) × 100] was calculated for the whole group and individually for sleep and nocturnal HRV indices across the 14 days.

Linear mixed model (lmm) and generalized linear mixed model (glmm) analysis were performed to examine differences in sleep and nocturnal HRV indices, training and match load parameters and perceived ratings of wellbeing across the 14 days of data collection. An α-level of 0.05 was set as the level of significance for statistical comparisons. The days with training sessions and matches were included as a fixed effect and player identity (subject ID) as the random effect. The variance-covariance structures were selected according to the smallest Akaike Information Criterion. Bonferroni pairwise comparisons were used to show the day-to-day mean differences for sleep and nocturnal HRV indices, training and match load parameters and perceived ratings of wellbeing.

We tested within-subjects correlations (r, 95% CI) (Bland and Altman, 1995) between sleep and nocturnal HRV indices with training and match load parameters. We qualitatively interpreted the magnitudes of correlation using the following criteria: *trivial* (*r* ≤ 0.1), *small* (*r* = 0.1–0.3), *moderate* (*r* = 0.3–0.5), *large* (*r* = 0.5–0.7), *very large* (*r* = 0.7–0.9), and *almost perfect* (*r* ≥ 0.9) (Hopkins et al., 2009). When the 95% CI overlapped positive and negative values, the effect was deemed to be *unclear*.

Lmm and glmm statistical analyses were conducted using SPSS software (version 27.0.1, SPSS Inc., Chicago, IL, United States) and for within-subjects correlation was used a rmcorr package in R statistical software (version 3.4.1, R Foundation for Statistical Computing, Vienna, Austria).

## Results

Actigraphy sleep and nocturnal HRV indices, training and matches load parameters and perceived ratings of wellbeing per day are summarized in Table 1. Training and match load were significantly higher in both MD_1_ and MD_2_ when compared with each ET. The fourth evening-time training session (ET_4_) had the lowest average TRIMP and s-RPE, while the highest TRIMP and s-RPE were recorded in MD_1_.

**TABLE 1:** Players actigraphy sleep and nocturnal heart rate variability indices, training and match load parameters and perceived ratings of wellbeing during the 14 days of in-season competitive phase (*n* = 34).

	Sleep duration (h)	Sleep efficiency (%)	lnRMSSD (ms)	lnLF (ms^2^)	lnHF (ms^2^)	HR_*ex*_ (bpm)	HR_peak_ (%)	TRIMP (AU)	s-RPE (AU)	Wellbeing (AU)
ET_1_	6.8*^#†‡⋄⊕∴^ (6.5; 7.2)	87 (85; 90)	4.2 (4.0; 4.4)	6.3 (5.8; 6.7)	7.3 (6.8; 7.7)	145*^#^ (141; 149)	75*^#^ (73; 77)	188*^#^ (169; 209)	397*^#^ (354; 446)	9.8 (8.8; 10.8)
RD_1_	8.3 (8.0; 9.3)	88 (86; 90)	4.3 (4.1; 4.5)	6.2 (5.8; 6.7)	7.4 (6.9; 7.8)					
ET_2_	7.0*^#†‡⋄⊕∴^ (6.6;7.3)	86 (84; 88)	4.2 (4.0; 4.4)	6.3 (5.8; 6.6)	7.3 (6.9; 7.7)	145*^#^ (141; 149)	76*^#^ (74; 78)	189*^#^ (170; 210)	411*^#^ (366; 461)	10 (8.1; 10.1)
RD_2_	8.4 (8.0; 8.8)	88 (86; 91)	4.4 (4.1; 4.6)	6.2 (5.9; 6.7)	7.5 (7.1; 7.9)					
ET_3_	6.5*^#†‡⋄⊕∴^ (6.2; 6.9)	87 (86; 99)	4.3 (4.0; 4.5)	6.7 (6.2; 7.1)	7.4 (7.0; 7.8)	143*^#^ (139; 147)	74*^#^ (72; 77)	190*^#^ (171;211)	403*^#^ (359; 451)	9.1 (7.6; 9.6)
RD_3_	7.8*^#^ (7.4; 8.1)	88 (85; 91)	4.3 (4.1; 4.5)	6.4 (6.0; 6.9)	7.4 (7.0; 7.9)					
MD_1_	8.7 (8.2; 9.2)	86 (83; 89)	4.2 (3.9; 4.4)	6.5 (6.0; 7.1)	7.2 (6.7; 7.6)	158 (152; 163)	82 (79; 84)	257 (227; 291)	686 (597; 789)	8.6 (7.0; 9.5)
ET_4_	6.9*^#†‡⋄⊕∴^ (6.7; 7.4)	90 (88; 92)	4.2 (4.0; 4.4)	6.2 (5.8; 6.7)	7.1 (6.7; 7.5)	146*^#^ (141; 150)	76*^#^ (74; 78)	176*^#^ (158; 196)	345*^#^ (308; 387)	9.9 (8.9; 10.9)
RD_4_	8.4 (8.0;8.8)	90 (88; 93)	4.4 (4.1; 4.6)	6.1 (5.8; 6.7)	7.3 (6.9; 7.7)					
ET_5_	6.7*^#†‡⋄⊕∴^ (6.5; 7.0)	88 (86; 90)	4.1 (3.9; 4.3)	6.3 (5.8; 6.7)	7.0 (6.7; 7.4)	147*^#^ (143; 151)	77*^#^ (75; 79)	187*^#^ (168; 208)	383*^#^ (341; 429)	9.3 (8.3; 10.3)
RD_5_	8.1 (7.8; 8.5)	89 (87; 91)	4.4 (4.2; 4.6)	6.2 (5.9; 6.7)	7.4 (7.0; 7.8)					
ET_6_	6.8*^#†‡⋄⊕∴^ (6.5; 7.2)	88 (86; 90)	4.2 (3.9; 4.4)	6.4 (5.8; 6.7)	7.1 (6.7; 7.5)	144*^#^ (139; 148)	75*^#^ (73; 77)	188*^#^ (169; 209)	377*^#^ (336; 423)	8.9 (7.9; 9.9)
RD_6_	8.0 (7.6; 8.4)	89 (87; 91)	4.4 (4.1; 4.6)	6.2 (6.0; 6.9)	7.4 (7.0; 7.8)					
MD_2_	8.8 (8.3; 9.5)	87 (84; 89)	4.2 (4.0; 4.5)	6.5 (6.0; 7.1)	7.2 (6.8; 7.7)	156 (151; 161)	81 (78; 83)	243 (215; 276)	619 (539; 711)	8.8 (7.2; 9.6)

Values are group mean and 95% confidence interval estimates.

* Significantly different from MD_1_. ^#^Significantly different from MD_2_. ^†^Significantly different from RD_1_. ^‡^Significantly different from RD_2_. ^⋄^Significantly different from RD_3_. ^⊕^Significantly different from RD_4_. ^∅^Significantly different from RD_5._^∴^Significantly different from RD_6_. ET, evening-time training sessions; RD, rest-days; MD, match-days; lnRMSSD, logarithm of the root mean square of successive R-R intervals; lnLF, natural logarithm of low frequency; lnHF, natural logarithm of high frequency; HR_*ex*_, the average heart rate during exercise; HR_peak_, peak heart rate; TRIMP, training impulse; s-RPE, session-rating of perceived exertion; AU, arbitrary units.

On average, sleep duration ranged between 6.5 (6.2; 6.9) to 8.8 h (8.3; 9.5), and sleep efficiency ranged between 86 (83; 89) to 90% (88; 93). Sleep duration was significantly reduced after each ET compared to RD and both MD (Table 1). The third evening-time training session (ET_3_) had the lowest average sleep duration, while the highest sleep duration was recorded in the MD_2_.

On average, lnRMSSD ranged between 4.1 (3.9; 4.3) to 4.4 ln[ms] (4.1; 4.6), lnLF ranged between 6.3 (5.9; 6.7) to 6.7 ln[ms^2^] (6.3; 7.1) and lnHF ranged between 7.2 (6.8; 7.6) to 7.5 ln[ms^2^] (7.1; 7.9).

No differences in sleep efficiency, nocturnal HRV indices and perceived ratings of wellbeing were found between the 14 days.

Due to technical problems and/or lack of player compliance, we had the following missing data: sleep and HRV indices *n* = 28 (6%), workload load parameters and perceived ratings of wellbeing *n* = 232 (49%).

**FIGURE 2:**
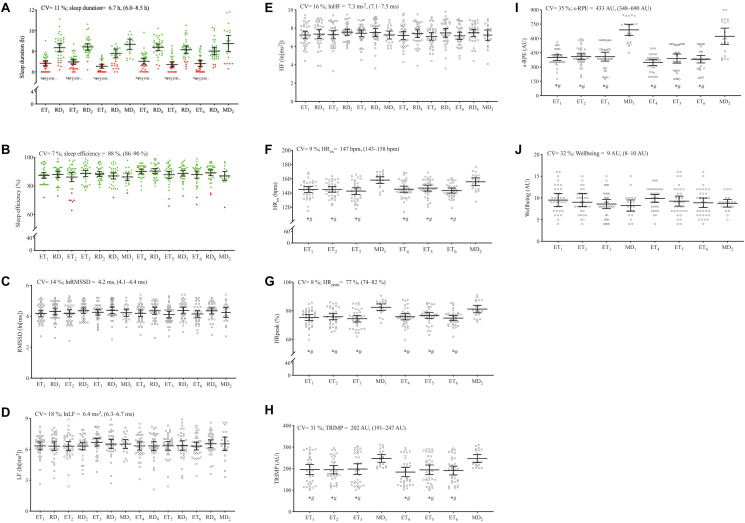
Descriptive group data and individual (*n* = 34) responsiveness for sleep **(A,B)** and nocturnal HRV indices **(C–E)**, training and match load parameters **(F–I)**, and perceived ratings of wellbeing **(J)** during 14 days of in-season competitive phase in high-level female soccer players. Horizontal black lines show group mean ± 95% confidence interval for each day. Coefficient of variation (CV), averages, maximum, and minimum values are also presented. Graph **(A)** and **(B)**: The red dots represent the days where sleep duration and sleep efficiency were lower than the recommended amounts (i.e., sleep duration < 7 h and sleep efficiency < 75%), respectively. The green dots represent the days where sleep duration and sleep efficiency were considered as recommended. *Significantly different from MD_1_. ^#^Significantly different from MD_2_. ^†^Significantly different from RD_1_. ^‡^Significantly different from RD_2_. ^⋄^Significantly different from RD_3_. ^⊕^Significantly different from RD_4_. ^∅^Significantly different from RD_5._^∴^Significantly different from RD_6._ ET, evening-time training sessions; RD, rest-days; MD, match-days; lnRMSSD, logarithm of the root mean square of successive R-R intervals; lnLF, natural logarithm of low frequency; lnHF, natural logarithm of high frequency; HR_*ex*_, the average heart rate during exercise; HR_peak_, peak heart rate; TRIMP, training impulse; s-RPE, session-rating of perceived exertion; AU, arbitrary units.

Figure 2 displays the group and individual sleep and nocturnal HRV indices, training, and match load parameters and perceived ratings of wellbeing data for each player.

Sleep duration CV ranged between 9 and 22%, while sleep efficiency ranged between 2 and 11% across the 14 days. The recommended cut-point of 7 h/night was not always reached by several players, especially after ET. Regarding sleep efficiency, only a limited number of players had episodes of sleep efficiency ≤ 74%.

Nocturnal HRV indices were stable during the 14-day period, while individual lnRMSSD, lnLF, and lnHF CV ranged between 3–23%, 5–46%, and 4–29%, respectively. Higher CV of lnRMSSD, lnLF and lnHF were particularly observed in four players (player 12: 16, 19, and 20%; player 24: 15, 19, and 16%; player 25: 23, 46, and 29%; and player 27: 15, 22, and 18%; respectively).

**FIGURE 3:**
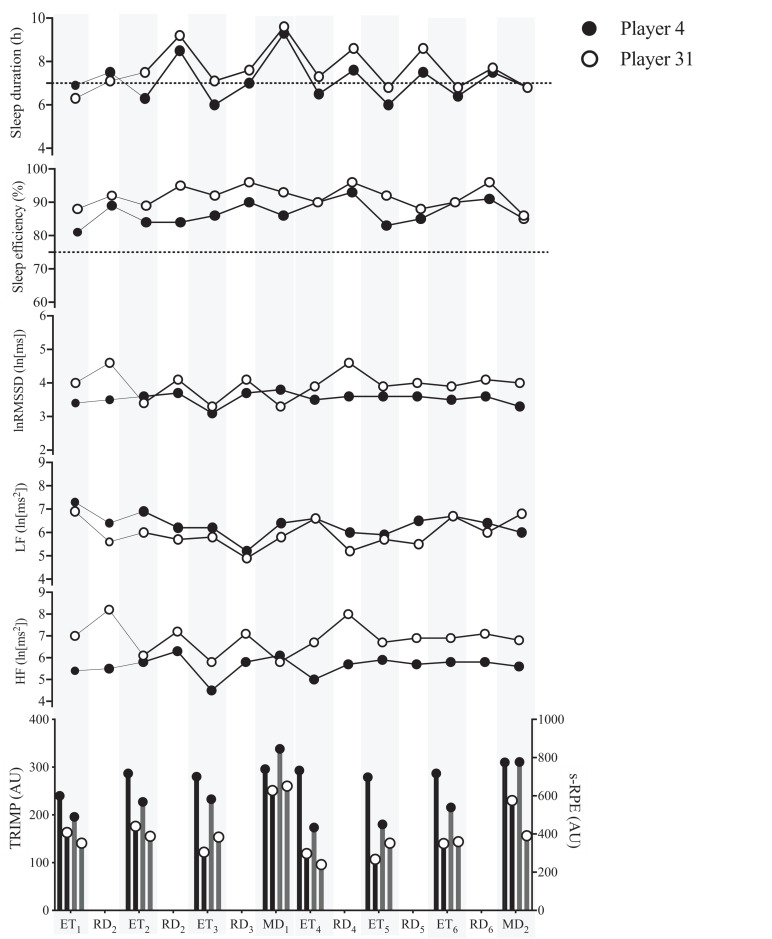
Sleep and nocturnal HRV indices of two players, that played on both match-days (MD), with the smallest (player 31) and highest (player 4) values of training impulse (TRIMP) and session-rating of perceived exertion (s-RPE), accumulated during the 14 days. The black line dashes represent sleep duration and sleep efficiency recommended amounts (i.e., sleep duration < 7 h and sleep efficiency < 75%), respectively. Black and gray bars represent accumulated TRIMP and s-RPE, respectively. Shaded areas denote ET and MD. ET, evening-time training sessions; RD, rest-days; lnRMSSD, logarithm of the root mean square of successive R-R intervals; lnLF, natural logarithm of low frequency; lnHF, natural logarithm of high frequency; AU, arbitrary units.

Figure 3 illustrates the sleep and nocturnal HRV indices of two players that played on both MD, representing the lowest and highest values of accumulated TRIMP (player 31:1,308 AU and player 4:2,272 AU) and s-RPE (player 31:3,118 AU and player 4:4,686 AU), during the 14 days.

The within-subject correlations of sleep and nocturnal HRV indices with training and match load parameters during the 14 days analysis are presented in Table 2. A *small* negative correlation was found between TRIMP and sleep duration and sleep efficiency. A *moderate* and *small* negative correlation was found between s-RPE and sleep duration and sleep efficiency.

**TABLE 2:** Within-subject correlation between sleep and nocturnal heart rate variability indices with session-rating of perceived exertion (s-RPE) and training impulse (TRIMP) during the 14 days of in-season competitive phase (*n* = 34).

	s-RPE			TRIMP		
		
	*r* (95% Confidence Interval)	*P*	Description	*r* (95% Confidence Interval)	*P*	Description
Sleep duration	−0.43 (−0.53; −0.32)	<0.001	Moderate	−0.25 (−0.36; −0.12)	<0.001	Small
Sleep efficiency	−0.17 (−0.29; −0.05)	0.02	Small	−0.20 (−0.32; −0.08)	0.004	Small
lnRMSSD	0.07 (−0.05; 0.20)	0.38	Unclear	0.01 (−0.12; 0.14)	0.97	Unclear
lnLF	0.09 (−0.04; 0.21)	0.26	Unclear	0.05 (−0.07; 0.18)	0.53	Unclear
lnHF	0.01 (−0.12; 0.14)	0.97	Unclear	−0.02 (−0.14; 0.11)	0.92	Unclear

*lnRMSSD, natural logarithm of square root of the mean of the sum of the squares of differences between adjacent NN intervals; lnLF, natural logarithm of low-frequency; lnHF, natural logarithm of high-frequency; LF/HF, ratio of the low to high frequency power.*

## Discussion

The study followed an observational design, highlighting individual sleep habits and nocturnal HRV responses, and the intra-individual associations of training and match workload with sleep and nocturnal HRV indices in high-level female soccer players throughout a 2-week in-season competitive period. We found that after ET a higher number of players slept less than 7 h/night in contrast to the remaining days. Only 1–6 players had a sleep efficiency < 75%/night, especially after ET_3_ (*n* = 6) in contrast to the remaining days.

Another important finding was the negative association between training and match workload and sleep indices. Finally, the training and matches workload did not affect nocturnal HRV, demonstrated by the absence of intra-individual associations with nocturnal HRV indices. However, individually, four players appeared to present higher lnRMSSD CV, lnLF CV, and lnLF CV compared to the remaining players. Therefore, our hypothesis was only partially confirmed, as only the sleep indices were negatively associated with training and match workloads.

In the current study, an important finding was that sleep duration was significantly reduced following ET compared to MD and RD. Also, sleep duration was generally below than that recommend by the National Sleep Foundation (Hirshkowitz et al., 2015; Ohayon et al., 2017) and more recently by the Sleep Consensus Recommendations for athletes (Walsh et al., 2020). This is consistent with recent studies showing a reduction in sleep duration following ET compared to RD and/or MD in high-level female soccer players (Costa et al., 2018a,b). Evening-training starting times might be a key factor that could negatively affect sleep variables in athletes. The impact of night schedule time on subsequent sleep was confirmed elsewhere (Sargent et al., 2014). This finding is also corroborated by the observed *small* to *moderate* negative within-subject correlations found between training and match workload and sleep indices. Notably, the lowest and the highest workloads appeared to have resulted in alterations on sleep durations during the competitive 2-week period. Periods of intensified training loads have been associated with higher sleep disruption (Hausswirth et al., 2014). This occurrence may be a result of overreaching (Hausswirth et al., 2014) and pro-inflammatory responses (Irwin et al., 2016). Similarly, high training loads (such as soccer match) are also likely to induce similar physiological responses (e.g., muscle damage and elevated inflammatory markers) (Mohr et al., 2016). Nevertheless, further research is required to determine the physiological mechanisms underlying the relationships (workload and sleep) and how they may impact recovery and performance.

In the present study, no significant nocturnal HRV changes were noticed during ET compared with the MD and RD, and *unclear* within-subject correlations with training and match load were noted. This is also consistent with recent studies conducted with high-level (Costa et al., 2018b,c) and elite (Costa et al., 2019a) female soccer players. This findings may indicate that the used HRV-derived method (i.e., SWSE method) was not sensitive enough to detect overnight cardiac autonomic activity disturbances (Costa et al., 2018c). Alternatively, it could also be suggested that the amount of training and match workload prescribed to the players was not high enough to cause meaningful changes in cardiac sympathetic and parasympathetic activities. In fact, the lowest and the highest workloads did not appear to have caused different vagally mediated responses during the competitive 2-week period. Supporting this notion, the perceived wellbeing rating [as established by Hooper et al. (1995)] of the players did not change across the ET and MD. Nonetheless, this topic deserves future studies. For the moment, in highly trained female soccer players, training and match workloads seem not to induce significant changes in HRV post-exercise night sleep throughout a 2-week in-season competitive period.

The lnRMSSD CV has been assessed in studies involving highly-trained athletes as a marker of variation in daily assessed lnRMSSD (Esco et al., 2014). In one study (Flatt et al., 2017a), the authors suggested that a high diurnal lnRMSSD CV was positively associated with perceived fatigue and negatively associated with the physical fitness of female soccer players. Moreover, another study found that diurnal lnRMSSD CV measured in swimmers can increase to values > 10% during overload periods (Flatt et al., 2017b). In the present study, as a group, lnRMSSD derived from the SWSE method displayed high average CV (14%). Moreover, individually, four players (player 12, 24, 25, and 27) presented higher lnRMSSD CV and reduced lnRMSSD, contrasting with the remaining players. Thus, nocturnal HRV was more susceptible to variations in these four players. Furthermore, it could be speculated that higher lnRMSSD CV linked with reduced average lnRMSSD during training and matches may be interpreted as a sign of overreaching (Flatt et al., 2017a). On the other hand, a higher resilience to sustained elevated training and match workloads without presenting signs of severe nocturnal cardiac autonomic perturbation, and a higher readiness to perform (Esco et al., 2014) may, in part, explain these results. Therefore, although no significant within-subject correlation could be detected between HRV indices and training and match workload, it is highly advisable that coaches closely examine individual players that may be suffering some detrimental effects during the competition, in order to promote their recovery through sleep hygiene and other means (Simpson et al., 2017). In fact, in a recent study conducted in non-professional male soccer teams, the authors explored the effect of an acute sleep hygiene strategy following a late-evening soccer-specific session (Vitale et al., 2019). They found that soccer players may benefit from acute sleep hygiene strategies to reduce the time to sleep onset after late-evening training sessions and therefore a more restorative sleep with a reduction of the stress imposed by the training session with a reduced cortisol awakening response.

Considering the nature of this observational/descriptive study, findings should be interpreted in light of the study limitations. Some potential factors that could have influenced both sleep duration and quality were not controlled, such as changes in hormonal levels, food intake before bedtime, naptime, level of light exposure during daytime and use of electronic devices. Unfortunately, technical problems and/or player compliance lead (i.e., most of the player’s wasn’t enough time to complete the diary and/or just lost interest) to several missing data points (i.e., diaries without answers), and for this reason, we decided to remove sleep diary information from the study. Finally, other potential limitations are the possible influence of missing data on the presented CV values and the lack of a time-point with no training or matches (baseline). However, this observational study was set in a real-world scenario, which limits the access to some of those measurements. Moreover, this is a longitudinal study design with individual and within-subjects analysis of data collected with wrist-worn accelerometers and HR monitors previously validated against polysomnography (Sargent et al., 2016) and standard electrocardiogram equipment to detect heartbeats (Parak and Korhonen, 2015), respectively. Nevertheless, the optimal amount of sleep on an individual basis may be difficult to establish (Watson, 2017), but at a minimum, adult and youth athletes who demonstrate an average sleep of less than 7 or 8 h, respectively, likely warrant additional evaluation to identify their specific sleep barriers.

It is also important to recognize the methods applied in the current study as a strength, especially for the practical applications that should be considered by practitioners in the field. Together, actigraphic variables and HRV indices provide more complete information on sleep patterns and cardiac autonomic function that elucidates about athlete recovery state. Moreover, both indices can be used to improve the quality of monitoring training and match load, and can be easily assessed at the training or match facilities and at the athletes’ own homes. Moreover, the methods can be implemented in a team’s daily routine. Finally, the individual variability in sleep and HRV observed on this study suggest the adoption of an individual approach to sleep (e.g., sleep hygiene), load monitoring, and recovery interventions in team sports. In fact, the competition scenario of successive matches often results in squad rotation between matches. However, some players will often be required to play in multiple matches. In these scenarios, individualized recovery and monitoring strategies may be required to ensure that all players are in peak condition for each match.

## Conclusion

The present observational study is the first to systematically analyze, simultaneously, consistent individual sleep and nocturnal HRV indices, and to explore the intra-individual associations of training and match workloads with sleep and nocturnal HRV indices in high-level female soccer players during an in-season competitive period. Overall, this study highlights the individual variability of sleep and nocturnal HRV indices, indicating that sleep duration may be affected by training and match schedules and workloads. Training and matches workload were not associated with nocturnal HRV in high-level female soccer players.

## Data Availability Statement

The raw data supporting the conclusions of this article will be made available by the authors, without undue reservation.

## Ethics Statement

The studies involving human participants were reviewed and approved by the study design was carefully explained to the participants, and written informed consent was obtained. The study followed the Declaration of Helsinki and was approved by the Ethics Committee of the Faculty of Sports, University of Porto (CEFADE 03.2017). The patients/participants provided their written informed consent to participate in this study.

## Author Contributions

JC, JB, and PF: conceptualization. JC, JB, PF, and FN: methodology and investigation. JC: software, writing—original draft preparation, and visualization. JC and PF: formal analysis and data curation. JB and AR: resources, supervision, and project administration. JB, PF, and AR: writing—review and editing. All authors have read and agreed to the published version of the manuscript.

## Conflict of Interest

The authors declare that the research was conducted in the absence of any commercial or financial relationships that could be construed as a potential conflict of interest. The handling editor declared a past co-authorship with the authors PF and JB.
